# Diagnostic work-up and systemic treatment for advanced non-squamous non-small-cell lung cancer in four Southeast Asian countries

**DOI:** 10.1016/j.esmoop.2022.100560

**Published:** 2022-08-18

**Authors:** R. Soo, L. Mery, A. Bardot, R. Kanesvaran, T.C. Keong, D. Pongnikorn, N. Prasongsook, S.H. Hutajulu, C. Irawan, A. Ab Manan, M. Thiagarajan, P. Sripan, S. Peters, H. Storm, F. Bray, R. Stahel

**Affiliations:** 1 Department of Hematology-Oncology, National University Hospital, Singapore, Singapore; 2 Section of Cancer Surveillance, International Agency for Research on Cancer, Lyon, France; 3 Division of Medical Oncology, National Cancer Centre, Singapore, Singapore; 4 Cancer Registry Unit, Lampang Cancer Hospital, Lampang, Thailand; 5 Medical Oncology Division, Department of Internal Medicine, Phramongkutklao Hospital, Bangkok, Thailand; 6 Division of Hematology and Medical Oncology, Department of Internal Medicine, Faculty of Medicine, Public Health and Nursing, Universitas Gadjah Mada/Dr. Sardjito General Hospital, Yogyakarta, Indonesia; 7 Division of Hematology and Medical Oncology, Department of Internal Medicine, Faculty of Medicine, Universitas Indonesia/Dr. Cipto Mangunkusumo General Hospital, Jakarta, Indonesia; 8 Malaysian National Cancer Registry Department, National Cancer Institute, Ministry of Health Malaysia, Putrajaya, Malaysia; 9 Department of Radiotherapy and Oncology, Kuala Lumpur Hospital, Kuala Lumpur, Malaysia; 10 Research Institute for Health Sciences, Chiang Mai University, Chiangmai, Thailand; 11 Department of Oncology, Centre Hospitalier Universitaire Vaudois, Lausanne, Switzerland; 12 Danish Cancer Society, Copenhagen, Denmark;; 13 ETOP IBCSG Partners Foundation, Bern, Switzerland

**Keywords:** molecular testing, Asia, population-based cancer registry, lung cancer, NSCLC

## Abstract

**Background:**

Lung cancer is the second most common cancer and leading cause of cancer mortality worldwide. Recent advances in molecular testing and targeted therapy have improved survival among patients with metastatic non-small-cell lung cancer (NSCLC). We sought to quantify and describe molecular testing among metastatic non-squamous NSCLC cases in selected Southeast Asian countries and describe first-line therapy chosen.

**Patients and methods:**

A retrospective study was conducted based on incident lung cancer cases diagnosed between 2017 and 2019 in Lampang (Thailand), Penang (Malaysia), Singapore and Yogyakarta (Indonesia). Cases (*n* = 3413) were defined using the International Classification of Diseases for Oncology third edition. In Singapore, a clinical series obtained from the National Cancer Centre was used to identify patients, while corresponding population-based cancer registries were used elsewhere. Tumor and clinical information were abstracted by chart review according to a predefined study protocol. Molecular testing of epidermal growth factor receptor (EGFR), anaplastic lymphoma kinase (ALK) gene rearrangement, *ROS1* gene rearrangement and *BRAF* V600 mutation was recorded.

**Results:**

Among 2962 cases with a specified pathological diagnosis (86.8%), most patients had non-squamous NSCLC (75.8%). For cases with staging information (92.1%), the majority presented with metastatic disease (71.3%). Overall, molecular testing rates in the 1528 patients with stage IV non-squamous NSCLC were 67.0% for EGFR, 42.3% for ALK, 39.1% for ROS1, 7.8% for BRAF and 36.1% for PD-L1. Among these patients, first-line systemic treatment included chemotherapy (25.9%), targeted therapy (35.6%) and immunotherapy (5.9%), with 31% of patients having no record of antitumor treatment. Molecular testing and the proportion of patients receiving treatment were highly heterogenous between the regions.

**Conclusions:**

This first analysis of data from a clinically annotated registry for lung cancer from four settings in Southeast Asia has demonstrated the feasibility of integrating clinical data within population-based cancer registries. Our study results identify areas where further development could improve patient access to optimal treatment.

## Highlights

• Clinically annotated registry data of lung cancer patients from Lampang, Penang, Singapore, and Yogyakarta.
• Three thousand four hundred thirteen patients diagnosed between 2017 and 2019.
• Thousand five hundred twenty-eight patients with stage IV non-squamous NSCLCEGFR testing performed in 67%, ranging from 14% to 89% according to region.
• EGFR mutations identified in 58% with little differences between the regions.

## Introduction

According to global estimates in 2020, lung cancer was the second most commonly diagnosed cancer, accounting for 11% of the total new cancer cases, and the leading cause of cancer death, equivalent to 18% of total cancer deaths.^1^ Of the 2.2 million patients newly diagnosed with lung cancer, 60% were from Asia, and of the 1.8 million who died due to lung cancer, 62% were from Asia. In Southeast Asia, the age-standardized incidence rate is estimated to be 26.4 per 100 000 in males and 9.6 per 100 000 in females.^2^

Over the past decade, treatment options for patients with metastatic non-small-cell lung cancer (NSCLC) have changed dramatically. Previously, platinum-based combination therapy was the mainstay of treatment for patients with metastatic disease^3^ with an improvement in survival compared with best supportive care, while comparative studies of different platinum-based treatments failed to identify the superiority of a particular combination.^4^^,^^5^ Subsequently for patients with non-squamous NSCLC, a small, but significant, survival benefit was demonstrated by the addition of bevacizumab to chemotherapy provided^6^^,^^7^ or the use of pemetrexed combination instead of gemcitabine combinations.^8^ The situation changed dramatically with the advent of targeted therapy based on the determination of oncogenic driver mutations, including activating epidermal growth factor receptor (EGFR) mutations and anaplastic lymphoma kinase (ALK) rearrangement, and currently over 10 molecular targets have been identified.^9^, ^10^, ^11^ Currently, the median survival time for patients with *EGFR*-mutated NSCLC is in the range of 2-4 years^12^^,^^13^ and for patients with *ALK*-rearranged NSCLC, over 7 years.^12^^,^^14^^,^^15^

For patients with NSCLC without oncogenic driver mutations, a further leap in therapeutic advances was the introduction of immune checkpoint inhibitors. Immune checkpoint inhibition alone or in combination with histology-specific chemotherapy is currently recommended for most patients whose tumor lack oncogenic driver alterations.^9^, ^10^, ^11^

The Evaluating Medical Oncology Outcomes (EMOO) in Asia study was designed to establish a clinical annotated population-based cancer registry in a collaboration between the European Society of Medical Oncology (ESMO), the International Agency for Research on Cancer (IARC) and partner institutions in Indonesia, Malaysia, Singapore and Thailand. More specifically, the study aimed to examine lung cancer incidence, alongside diagnostic and clinical information and outcomes for patients diagnosed in the years 2017-2019. The focus of this manuscript is a population-based assessment of the diagnostic work-up of the cases captured, the extent of molecular testing carried out and the use of targeted treatment for patients with advanced non-squamous NSCLC.

## Patients and methods

Indonesia, Malaysia, Singapore and Thailand were selected as the countries for the study following meetings with representatives from respective clinical oncology societies and cancer registries. In July 2019, a study protocol was developed to assess the feasibility of collecting clinical information on newly diagnosed lung cancer patients. Before the commencement of data collection, a 2-day in-person training workshop on the definitions and methods with representatives from each participating site was held in September 2019. Ethics approval for the study was obtained through the IARC’s institutional review board and locally from each participating site. Implementation of the study was overseen by local principal investigators and by an advisory committee comprising representatives from ESMO and IARC. Site visits were held to introduce the study to key stakeholders and finalize any required adjustments. Funding for the study was provided by ESMO.

We defined eligible cases as all primary lung cancer patients were diagnosed between 1 January 2017 and 31 December 2019 with an International Classification of Diseases for Oncology, third edition (ICD-O-3) code of C33.9, C34.0-C34.3 or C34.8-C34.9.^16^ Two methods were used as sources for case finding. The first involved subnational population-based cancer registries (PBCRs) in Lampang (Thailand), Penang (Malaysia) and Yogyakarta (Indonesia) to produce a listing of incident lung cancer cases among residents from each respective geographic area. The PBCR in Yogyakarta was newly established as part of this study, while Lampang and Penang are longstanding registries considered as high quality based on a periodic assessment of cancer registries from around the world at IARC, Cancer Incidence in Five Continents.^17^ The second method of case finding was specific to Singapore, where the national PBCR was not accessible to the study. Rather, inclusion was restricted to consenting patients participating in the Lung Cancer Consortium Singapore National Lung Cancer Research Study, an open-based clinical research platform.^18^ A request to the National Registry of Diseases Office in Singapore was made to obtain aggregate data during the study period from the Singapore Cancer Registry.

Chart reviews conducted by trained personnel from each PBCR were used to abstract the required clinical data elements. Vital status and cause of death were obtained by Lampang, Penang and Yogyakarta and linked to the PBCR, while aggregate mortality data were available in Singapore.

A set of indicators were developed using an iterative process led by the authors. An initial list was generated based on a review of published and gray literature.^19^, ^20^, ^21^, ^22^, ^23^, ^24^, ^25^, ^26^, ^27^, ^28^, ^29^, ^30^, ^31^, ^32^, ^33^, ^34^, ^35^, ^36^, ^37^, ^38^ The list was refined through clinical input from the study representatives to examine relevance and feasibility. A final set of 38 indicators were selected that were grouped into six major themes: (i) process and diagnostic; (ii) staging; (iii) treatment; (iv) outcomes; (v) comorbidity; and (vi) palliative care. Indicators for the study included many additional data items than those normally collected by a PBCR, necessitating chart reviews by trained staff to obtain the required clinical information. An online data collection tool was developed in English using REDCap^39^ to electronically capture information from two files—an incident file and a clinical file. The incident dataset, consisted of 19 fields that are commonly collected by PBCRs, such as patient demographics, diagnoses and tumor characteristics.^40^ The clinical dataset was designed to capture the remaining required data fields (*n* = 60).

The incidence and clinical files were transferred separately to IARC for centralized data quality review and analyses. Personal information on patients was removed at each center before the files were being sent to IARC. A unique study identification number was created in order to combine files and communicate with each site about queries of specific records. To help ensure the accuracy of the linkage, records were cross-checked through a common set of variables on each file—date of birth, date of diagnosis, sex and ICD-O topography and morphology diagnosis. Records with the same study identification number that had differing values on common fields were rechecked with each study site to correct potential mismatches. A merged study file was created and processed using the IARC-Check program to assess inconsistency in reporting.^41^ Results of the edit check were sent to each site asking that they correct errors before inclusion in the final analyses.

Results were grouped by histology. NSCLC was classified into squamous cell and non-squamous cell, with the latter further divided into adenocarcinoma, large cell carcinoma and ‘not otherwise specified’. Grouped TNM (tumor–node–metastasis) stage was captured as a field in data abstraction. When this was missing, stage was derived by combining the individual components of tumor, node and metastasis beginning with clinical elements followed by pathology. The UICC TNM seventh edition was used in Yogyakarta, while Penang and Singapore used the UICC TNM eighth edition to classify stage; Lampang used a mixture of the two editions and had three cases classified in the sixth edition.^42^ When neither was available, grouped TNM codes supplied by the cancer registry were used. *EGFR* and *BRAF* mutation, and *ALK* and *ROS1* fusion status from pre-treatment tumor samples were collected. Also captured were the use of next-generation sequencing (NGS) and the determination of PD-L1 expression by immunohistochemistry. A response of ‘unknown’ was given when no record of a test being administered was found in the patient’s chart. We reported molecular test results by grouping together ‘unknown’, ‘no test administered’ and ‘missing’ responses. PD-L1 test results were recorded as positive if the percentage of cells positive was greater or equal to one. Treatment data collected were limited to the first 6 months after diagnosis. Specific anticancer agents used for first-line therapy were collected based on a pre-established selected list of agents. For data fields with ordinal or nominal categories, frequencies and proportions were calculated.

## Results

Patient and tumor characteristics by region are presented in Table 1. A total of 3413 cases were recorded with the largest proportion from Singapore (39.2%, *n* = 1338), followed by Lampang (29.1%, *n* = 994), Yogyakarta (19.5%, *n* = 664) and Penang (12.2%, *n* = 417). Overall, for all sites excluding Singapore, the percentage of observed cases compared to those expected was 80.5% (*n* = 2650 expected cases). This percentage ranged from 54.5% in Penang in 2019 (118 cases: 217 expected), to 113.1% in Lampang in 2018 (377 observed cases: 333 expected). Across the years of diagnosis, we observed the lowest percentage of completeness in 2019 (75.1%), the final year of the study, in which data collection was most greatly impacted by coronavirus disease 2019 (COVID-19). Estimates of the number expected were derived a priori by each center, based on cases accrued in previous years.

**Table 1:** Patient and tumor characteristics by region

Characteristic	Region				
	Lampang	Penang	Singapore	Yogyakarta	Total
Cases, *n* (row %)	994 (29.1)	417 (12.2)	1338 (39.2)	664 (19.5)	3413
Year of diagnosis, *n* (%)					
2017	334 (33.6)	157 (37.6)	496 (37.1)	187 (28.2)	1174 (34.4)
2018	362 (36.4)	142 (34.1)	420 (31.4)	266 (40.1)	1190 (34.9)
2019	298 (30.0)	118 (28.3)	422 (31.5)	211 (31.8)	1049 (30.7)
Sex, *n* (%)					
Females	375 (37.7)	153 (36.7)	538 (40.2)	243 (36.6)	1309 (38.4)
Males	619 (62.3)	264 (63.3)	800 (59.8)	421 (63.4)	2104 (61.6)
Age at diagnosis, years, *n* (%)					
<55 years	115 (11.6)	80 (19.2)	181 (13.5)	179 (27.0)	555 (16.3)
55-64	265 (26.7)	116 (27.8)	359 (26.8)	216 (32.5)	956 (28.0)
65-74	324 (32.6)	137 (32.9)	478 (35.7)	167 (25.2)	1106 (32.4)
75-84	233 (23.4)	74 (17.7)	291 (21.7)	89 (13.4)	687 (20.1)
85+	57 (5.7)	10 (2.4)	29 (2.2)	13 (2.0)	109 (3.2)
Median age at diagnosis (range)	68 (25-97)	65 (23-93)	67 (19-93)	62 (20-94)	66 (19-97)
Smoking history, *n* (%)					
Current	135 (13.6)	91 (21.8)	419 (31.3)	69 (10.4)	714 (20.9)
Ever	405 (40.7)	138 (33.1)	250 (18.7)	143 (21.5)	936 (27.4)
Never	363 (36.5)	180 (43.2)	669 (50.0)	145 (21.8)	1357 (39.8)
Unknown	91 (9.2)	8 (1.9)	0 (0)	307 (46.2)	406 (11.9)
Basis of diagnosis, *n* (%)					
Histology	531 (53.4)	382 (91.6)	1175 (87.8)	76 (11.4)	2164 (63.4)
Cytology	97 (9.8)	4 (1.0)	152 (11.4)	535 (80.6)	788 (23.1)
Imaging	335 (33.7)	18 (4.3)	5 (0.4)	34 (5.1)	392 (11.5)
Clinical	31 (3.1)	13 (3.1)	2 (0.1)	19 (2.9)	65 (1.9)
Death certificate only	0 (0)	0 (0)	0 (0)	0 (0)	0 (0)
Unknown	0 (0)	0 (0)	4 (0.3)	0 (0)	4 (0.1)
Pathological diagnosis, *n* (%)					
Small-cell lung cancer	38 (3.8)	16 (3.8)	83 (6.2)	31 (4.7)	168 (4.9)
Non-small-cell lung cancer	558 (56.1)	337 (80.8)	1242 (92.8)	571 (86.0)	2708 (79.3)
Squamous cell carcinoma	85 (8.6)	63 (15.1)	164 (12.3)	151 (22.7)	463 (13.6)
Non-squamous cell carcinoma					
Adenocarcinoma	416 (41.9)	247 (59.2)	957 (71.5)	367 (55.3)	1,987 (58.2)
Large cell carcinoma	3 (0.3)	7 (1.7)	0 (0)	4 (0.6)	14 (0.4)
Non-small-cell lung cancer, NOS	54 (5.4)	20 (4.8)	121 (9.0)	49 (7.4)	244 (7.1)
Other	32 (3.2)	33 (7.9)	7 (0.5)	14 (2.1)	86 (2.5)
Lung cancer without a pathological diagnosis	366 (36.8)	31 (7.4)	6 (0.4)	48 (7.2)	451 (13.2)
Stage, grouped TNM, *n* (%)					
Stage I	32 (3.2)	20 (4.8)	273 (20.4)	0 (0)	325 (9.5)
Stage II	20 (2.0)	16 (3.8)	86 (6.4)	9 (1.4)	131 (3.8)
Stage III	129 (13.0)	89 (21.3)	200 (14.9)	27 (4.1)	445 (13.0)
Stage IV	735 (73.9)	266 (63.8)	764 (57.1)	476 (71.7)	2,241 (65.7)
Unknown	78 (7.8)	26 (6.2)	15 (1.1)	152 (22.9)	271 (7.9)
ECOG performance status, *n* (%)					
0	20 (2.0)	58 (13.9)	525 (39.2)	19 (2.9)	622 (18.2)
1	427 (43.0)	98 (23.5)	639 (47.8)	84 (12.7)	1,248 (36.6)
2	387 (38.9)	108 (25.9)	112 (8.4)	157 (23.6)	764 (22.4)
3	113 (11.4)	106 (25.4)	42 (3.1)	215 (32.4)	476 (13.9)
4	7 (0.7)	27 (6.5)	6 (0.4)	72 (10.8)	112 (3.3)
5	4 (0.4)	3 (0.7)	0 (0)	0 (0)	7 (0.2)
Unknown	36 (3.6)	17 (4.1)	14 (1.0)	117 (17.6)	184 (5.4)

ECOG, Eastern Cooperative Oncology Group; NOS, not otherwise specified; TNM, tumor–node–metastasis.

The median age at diagnosis was 66 years. Yogyakarta reported a lower median age of 62 years, driven by a higher proportion of patients under age 55 (27.0%) as compared to the other sites combined (13.7%). Male patients were more common overall (61.6%). Information on smoking status was available for most patients, with 39.8% reporting to have never smoked, while the remainder were current or former smokers.

Histology was the basis of diagnosis in 63.4% and cytology in 23.1% of cases. Histology was most common in Lampang, Penang and Singapore ranging from 53.4% to 91.6%, while cytology was most common in Yogyakarta (80.6%). SCLC was reported in 4.9% of cases (*n* = 168) and NSCLC in 79.3% of cases (*n* = 2708). Among NSCLC cases, 17.1% (*n* = 463) were squamous cell and 82.9% (*n* = 2245) were non-squamous NSCLC. Diagnosis was based uniquely on imaging or clinical judgment in 13.4% (*n* = 457) of patients, most commonly in Lampang (36.8% of patients), with the proportion between 0.5% and 8.0% in the other regions.

Stage IV disease, a focus of this manuscript, was recorded in 65.7% of patients (*n* = 2241). After excluding cases with an unknown stage, 71.3% of patients were diagnosed as stage IV. Of all patients, 1528 patients (44.8%) were diagnosed with stage IV non-squamous NSCLC (Table 2).

**Table 2:** Molecular testing results for stage IV NSCLC non-squamous patients by region

Molecular testing results	Region				Total
	Lampang	Penang	Singapore	Yogyakarta	
Cases (*N*)	374	184	647	323	1528
EGFR molecular mutation testing carried out, *n* (%)	52 (13.9)	109 (59.2)	626 (96.8)	237 (73.4)	1024 (67.0)
Positive	24 (46.2)	61 (56.0)	367 (58.6)	142 (59.9)	594 (58.0)
Deletion exon 19	15 (28.8)	37 (33.9)	178 (28.4)	73 (30.8)	303 (29.6)
Point mutation exon 21	6 (11.5)	21 (19.3)	148 (23.6)	31 (13.1)	206 (20.1)
Other	3 (5.8)	3 (2.8)	41 (6.5)	38 (16.0)	85 (8.3)
ALK gene rearrangement testing carried out, *n* (%)	23 (6.1)	46 (25.0)	577 (89.2)	1 (0.3)	647 (42.3)
Positive	5 (21.7)	5 (10.9)	40 (6.9)	1 (100.0)	51 (7.9)
ROS1 gene rearrangement testing carried out, *n* (%)	7 (1.9)	21 (11.4)	570 (88.1)	0 (0.0)	598 (39.1)
Positive	2 (28.6)	0 (0)	11 (1.9)	0 (0)	13 (2.2)
BRAF V600 mutation testing carried out, *n* (%)	1 (0.3)	5 (2.7)	113 (17.5)	0 (0.0)	119 (7.8)
Positive	0 (0)	0 (0)	1 (0.9)	0 (0)	1 (0.8)
PD-L1 immunohistochemistry testing carried out, *n* (%)	14 (3.7)	42 (22.8)	494 (76.4)	2 (0.6)	552 (36.1)
Positive^a^	6 (42.9)	25 (59.5)	344 (69.6)	1 (50.0)	376 (68.1)

ALK, anaplastic lymphoma kinase; EGFR, epidermal growth factor receptor; NSCLC, non-small-cell lung cancer; PD-L1, programmed death-ligand 1.

a PD-L1-positive immunohistochemistry ≥1% tumor cells.

The degree of molecular testing among patients with stage IV non-squamous NSCLC was heterogeneous between the regions (Table 2). Overall, molecular testing including EGFR, ALK, BRAFV600 or PD-L1 was carried out in 67% of 1045 histology specimens and 33% of 478 cytology specimens. *EGFR* mutations were tested in 67.0% of patients, with 13.9% in Lampang, 59.2% and 73.4% in Penang and Yogyakarta, respectively, and 96.8% in Singapore. Among the patients tested, the rate of *EGFR* mutations detected was 58.0%, with 51.0% (*n* = 303) of those being exon 19 deletions, 34.7% (*n* = 206) exon 21 point mutations and 14.3% (*n* = 85) other *EGFR* alterations. The proportion of other *EGFR* alterations was particularly high in Yogyakarta (16.0%).

Testing for *ALK* gene rearrangement was less frequent overall, except in Singapore where it was common (89.2%), followed by Penang (25.0%), with very low usage elsewhere. Positive tests results were seen in 7.9% of patients. Testing for *ROS1* gene rearrangement followed a similar pattern, being frequent in Singapore (88.1%), low in Penang (11.4%) and very low to absent elsewhere. Positive test results were seen in 2.2% of patients. Testing for *BRAF* V600E mutations was carried out overall in 7.8% of patients, with the majority from Singapore. The positive test rate for BRAF was 0.8%. PD-L1 followed a similar pattern, being tested in 76.4% and 22.8% of patients in Singapore and Penang, respectively, and seldom evaluated in the other two sites. Access to NGS was reported for 17.3% of patients from Singapore and 3.8% of patients from Penang.

First-line treatment is summarized for the 1528 stage IV non-squamous NSCLC patients in Table 3. Of these patients (*n* = 1528), 31.3% received no anticancer treatment. Of the patients receiving treatment (*n* = 1029), 52.9% received targeted therapy, 38.4% received chemotherapy and 8.7% received immunotherapy treatment. The distribution of treatment differed substantially between sites. In Lampang, 50.5% of patients received no treatment and among those who did receive treatment, it was mostly chemotherapy. In Penang and Yogyakarta, 41.8% and 42.7% received no treatment and the treatment administered was similar between chemotherapy and targeted treatment.

**Table 3:** Systemic therapy within 6 months post diagnosis for stage IV non-squamous NSCLC according to region

Treatment	Region				Total
	Lampang^a^	Penang^b^	Singapore^c^	Yogyakarta	
Cases (*N*)	374	184	647	323	1528
Chemotherapy, *n* (%)					
Yes	151 (40.4)	49 (26.6)	118 (18.2)	77 (23.8)	395 (25.9)
No	216 (57.8)	130 (70.7)	527 (81.5)	235 (72.8)	1108 (72.5)
Unknown	7 (1.9)	5 (2.7)	2 (0.3)	11 (3.4)	25 (1.6)
Targeted therapy, *n* (%)					
Yes	26 (7.0)	52 (28.3)	369 (57.0)	97 (30.0)	544 (35.6)
No	343 (91.7)	130 (70.7)	276 (42.7)	215 (66.6)	964 (63.1)
Unknown	5 (1.3)	2 (1.1)	2 (0.3)	11 (3.4)	20 (1.3)
Immunotherapy, *n* (%)					
Yes	3 (0.8)	4 (2.2)	83 (12.8)	0 (0)	90 (5.9)
No	363 (97.1)	174 (94.6)	562 (86.9)	309 (95.7)	1,408 (92.1)
Unknown	8 (2.1)	6 (3.3)	2 (0.3)	14 (4.3)	30 (2.0)
None, *n* (%)	189 (50.5)	77 (41.8)	75 (11.6)	138 (42.7)	479 (31.3)
Unknown treatment, *n* (%)	5 (1.3)	2 (1.1)	2 (0.3)	11 (3.4)	20 (1.3)

NSCLC, non-small-cell lung cancer.

a One case reported a combination of first-line chemotherapy and immunotherapy.

b One case reported a combination of first-line chemotherapy and targeted therapy, two cases reported a combination of chemotherapy and immunotherapy.

c One case reported a combination of first-line chemotherapy and targeted therapy, 41 cases reported a combination of chemotherapy and immunotherapy.

Details on targeted therapies were analyzed for 610 patients with stage IV adenocarcinoma with a known molecular status (Table 4). EGFR tyrosine kinase inhibitors (TKIs) were administered to 81.9% of the 558 patients with positive EGFR tests. First-, second- and third-generation EGFR TKIs were used in 57%, 22% and 21%, respectively. ALK TKIs were administered to 65.2% of the 46 ALK-positive with positive test and ROS1 TKIs to 30.8% of 13 ROS1 positivity. First-line immune checkpoint inhibitors were administered to 68 patients, and nearly all patients received pembrolizumab (97.1%, *n* = 66).

**Table 4:** First-line therapy among stage IV NSCLC adenocarcinoma patients with a positive molecular test result

Type of therapy	Number of patients	%
Molecular test		
Treatment administered		
First-line targeted therapy		
EGFR positive	558	
Targeted treatment administered^a^	457	81.9
Afatinib	99	21.7
Erlotinib	65	14.2
Gefitinib	197	43.1
Osimertinib	94	20.6
Other	14	3.1
No drug administered	101	18.1
ALK gene rearrangement	46	
Targeted treatment administered^b^	30	65.2
Alectinib	21	70.0
Ceritinib	4	13.3
Crizotinib	5	16.7
Other	2	6.7
No drug administered	16	34.8
ROS1 gene rearrangement	13	
Targeted treatment administered	4	30.8
Alectinib	0	0
Ceritinib	0	0
Crizotinib	4	100.0
Other	0	0
No drug administered	9	69.2

a 10 cases took two different drugs, one case took three different drugs.

b Two cases took two different drugs.

## Discussion

This is the first report on a clinical annotated population-based lung cancer registry of 3413 patients obtained from four different Southeast Asian settings. The EMOO in Asia study was designed to determine lung cancer incidence, diagnostic and clinical information and outcomes for patients diagnosed in Indonesia, Malaysia, Singapore and Thailand for the years 2017-2019. The focus of this manuscript is the diagnostic work-up, molecular testing and its translation into targeted therapy in patients with advanced non-squamous NSCLC.

A first finding that merits discussion is the difference in diagnostic approaches of lung cancer between regions. While this was based mainly on histology in Penang and Singapore, cytology was used most often in Yogyakarta. However, overall the diagnosis of lung cancer was based only on clinical examination or imaging in 13.4% of patients. This was most prevalent in the Lampang region at 36.8% and less often in Penang and Yogyakarta at 7.4% and 8.0% of patients, respectively. Reasons for the lack of diagnostic confirmation were not based on unavailable diagnostic methods, but rather the inability of patients to financially afford the procedures, or possibly, to a lesser degree, their poor general health, or late-stage disease, at presentation.^43^ The same reasons were suspected to account for the high proportion of over 40% of patients with metastatic NSCLC not receiving a systemic anticancer treatment after a diagnosis was made in Lampang, Penang and Yogyakarta.

To assess the use of molecular testing and targeted treatment, we focused on the 1528 patients with stage IV non-squamous NSCLC. Overall, the rate of molecular testing was highest for *EGFR* mutation at 67.0%. The rate of testing varied between the regions, being lowest in Lampang and highest in Singapore. During the study period, EGFR TKIs were available in Penang, Singapore and Yogyakarta; however, unaffordability was reported as obstacles to wider testing by Yogyakarta and Penang. In Lampang, EGFR TKIs were available only to Thai patients who were under the Civil Servant Medical Benefit Scheme.^44^ Testing rates in the same time frame were 75% for the United States based on the flatiron database and ranged from 73% to 85% in selected European countries.^45^^,^^46^

Testing for *ALK* gene rearrangement was reported as the next most commonly carried out followed closely by *ROS1* gene rearrangement testing. With the exception of Singapore, test rates for *ALK* gene rearrangement were low, ranging from 0.3% to 25% only. Among the three sites with lower proportions of testing, the reasons were unaffordability of performing the test itself and/or the unaffordability of ALK TKIs, other than for patients in Singapore.^47^, ^48^, ^49^, ^50^, ^51^

In patient populations where 40% or more are never smokers, molecular testing for an actionable molecular alteration is particularly important. Sixty percent of tumors tested harbored *EGFR* mutations. Of these, 82% received a targeted therapy which is certainly encouraging. However, we need to keep in mind that a proportion of patients did not have pathologic confirmation of their diagnosis and of those who did, the proportion of molecular testing carried out was low in some regions. Given the predominance of *EGFR*-mutated non-squamous NSCLC in this region, improvements of work-up leading to a pathological diagnosis and molecular EGFR testing, and subsequent affordable EGFR targeted therapy should be a priority.

In the study, two different methods of case finding were used. A subnational PBCR was the source in Lampang, Penang and Yogyakarta. Notwithstanding challenges due to COVID-19, a comparison of observed versus expected cases yielded a high degree of completeness. The unique features of a PBCR that include all cases in the targeted population, allowing for findings to be more easily generalized, have contributed to its importance in cancer control.^52^ While data from Singapore was from a single hospital and should be interpreted with caution, when compared with aggregated data obtained from the Singapore Cancer Registry, the distribution of cases was found to be similar. The high rates of testing and treatment patterns in Singapore may be in part due to their increased availability at the National Cancer Centre Singapore, while in the other settings, high patient costs were likely the main deterrent.^47^ In the case of Yogyakarta, the PBCR was newly established. Additional data quality assessments were conducted to ensure comparability of results with the other PBCRs.

In many low- and middle-income settings, cancer registries face challenges—in particular, the overall quality of medical records available, together with specific issues of access to private institutions.^53^ These challenges were largely overcome in this study through the selection of sites where co-operation with local data sources had been secured. To capture the movement of patients between private and public treatment and diagnostic centers, data from the PBCR are brought together to form one record for the patient. This aspect of PBCR lessens the likelihood of missing relevant information for the study when a patient is seen at multiple institutions during their care.

Data collection for the study was hampered by the emergence of the COVID-19 outbreak. A slow-down began in January 2020, culminating with a temporary stoppage of all data collection activities from March to May 2020 due to strict restrictions imposed on movement and outdoor activities. Cancer registries worldwide had similar experiences.^54^ Data collection resumed in June 2020 but remained limited as staff at centers had restrictions on work hours and where they could visit, as COVID-19 management became the priority. To compensate for the impact of COVID-19, centers were asked to prioritize data collection for 2017-2018 incident cases. Despite these limitations, the study was successful to gather real-word data from an estimated 80.5% of the expected lung cancer cases in the respective regions where PBCRs were used.

In a time where molecular testing is rapidly developing, in particular also with the introduction of NGS, and new molecular targets have become approved for targeted therapy, a limitation is the timeliness of our study based on medical records from 2017 to 2019. Nevertheless, a clear strength is the population basis of the PBCR data, enabling a description of the great heterogeneity of access to diagnosis, molecular testing and targeted treatment of non-squamous NSCLC in these four Southeast Asian settings that highlights where specific interventions could lead to improved patient care.

Our first analysis of the EMOO in Asia study demonstrates a great heterogeneity of access of patients with metastatic NSCLC to diagnostic work-up and advanced systemic treatment and highlights the importance to improve diagnostic testing rates for patients with NSCLC. This study has provided new evidence on lung cancer treatment and diagnostic work-up in diverse settings across Southeast Asia. The design has demonstrated that it is possible to obtain more detailed data than normally collected by PBCR but that approaches required flexibility to account for variation in the hospital systems within these areas. The findings call other cancer sites to be studied in light of the advances in biomarker-defined targeted treatments.

The need to address issues at the health systems level to improve outcomes in lower resource settings has been well documented.^55^ This disparity is a call for action for the communities to not only focus on implementing scientific advances, but also consider how we could act together to bring clinical advances to cancer patients at large, in particular situations such as EGFR mutations, where the prevalence in lung adenocarcinoma is 50% or higher. Global or societal initiatives calling for better access to cancer medicines and care are important,^56^ as is the World Health Organization essential medicines list (EML).^57^ Despite the acknowledgment that new cancer drugs are not equally valuable, the crisis in medication costs combined with disparities in wealth and resources objectively limit the optimization of the EML to more closely align it with best practice care.

The ESMO Magnitude of Clinical Benefit Scale is a metric of clinical benefit and can enhance the consistency and transparency of cancer drug evaluations.^58^ Rethinking drug accessibility, pricing and geographically defined reimbursement models is probably the most complex and important chapter on the cancer public policy agenda. Gathering real-world, population-based data such as in this and other studies and initiatives^59^, ^60^, ^61^, ^62^ are essential to document the reality and the needs, as well as to assess how such findings may serve to overcome the numerous challenges faced by the lung cancer community.

## References

[1] Sung H., Sung H., Ferlay J. (2021). Global Cancer Statistics 2020: GLOBOCAN estimates of incidence and mortality worldwide for 36 cancers in 185 countries. CA Cancer J Clin.

[2] Ferlay J. (2020). https://gco.iarc.fr/today.

[3] (1995). Chemotherapy in non-small cell lung cancer: a meta-analysis using updated data on individual patients from 52 randomised clinical trials. Non-small Cell Lung Cancer Collaborative Group. BMJ.

[4] NSCLC Meta-Analyses Collaborative Group (2008). Chemotherapy in addition to supportive care improves survival in advanced non-small-cell lung cancer: a systematic review and meta-analysis of individual patient data from 16 randomized controlled trials. J Clin Oncol.

[5] Schiller J.H., Harrington D., Belani C.P. (2002). Comparison of four chemotherapy regimens for advanced non-small-cell lung cancer. N Engl J Med.

[6] Sandler A., Gray R., Perry M.C. (2006). Paclitaxel-carboplatin alone or with bevacizumab for non-small-cell lung cancer. N Engl J Med.

[7] Zhou C., Wu Y.L., Chen G. (2015). BEYOND: a randomized, double-blind, placebo-controlled, multicenter, phase III study of first-line carboplatin/paclitaxel plus bevacizumab or placebo in Chinese patients with advanced or recurrent nonsquamous non-small-cell lung cancer. J Clin Oncol.

[8] Scagliotti G.V., Parikh P., von Pawel J. (2008). Phase III study comparing cisplatin plus gemcitabine with cisplatin plus pemetrexed in chemotherapy-naive patients with advanced-stage non-small-cell lung cancer. J Clin Oncol.

[9] The ESMO Guidelines Committee, Clinical Practice Living Guidelines *–* Metastatic Non-Small-Cell Lung Cancer | ESMO. 2020, European Society for Medical Oncology.

[10] Planchard D., Popat S., Kerr K. (2018). Metastatic non-small cell lung cancer: ESMO Clinical Practice Guidelines for diagnosis, treatment and follow-up. Ann Oncol.

[11] Novello S., Barlesi F., Califano R. (2016). Metastatic non-small-cell lung cancer: ESMO Clinical Practice Guidelines for diagnosis, treatment and follow-up. Ann Oncol.

[12] Ramalingam S.S., Vansteenkiste J., Planchard D. (2019). Overall survival with osimertinib in untreated, EGFR-mutated advanced NSCLC. N Engl J Med.

[13] Maemondo M., Fukuhara T., Saito H. (2020). NEJ026: final overall survival analysis of bevacizumab plus erlotinib treatment for NSCLC patients harboring activating EGFR-mutations. J Clin Oncol.

[14] Mok T., Camidge D.R., Gadgeel S.M. (2020). Updated overall survival and final progression-free survival data for patients with treatment-naive advanced ALK-positive non-small-cell lung cancer in the ALEX study. Ann Oncol.

[15] Pacheco J.M., Gao D., Smith D. (2019). Natural history and factors associated with overall survival in stage IV ALK-rearranged non-small cell lung cancer. J Thorac Oncol.

[16] World Health Organization (2013).

[17] Bray F., Ferlay J., Laversanne M. (2021).

[18] Tan A.C., Lai G.G.Y., Tan G.S. (2020). Utility of incorporating next-generation sequencing (NGS) in an Asian non-small cell lung cancer (NSCLC) population: incremental yield of actionable alterations and cost-effectiveness analysis. Lung Cancer.

[19] Aguilar K.M., Winfree K.B., Muehlenbein C.E. (2018). Treatment patterns by EGFR mutation status in non-small cell lung cancer patients in the USA: a retrospective database analysis. Adv Ther.

[20] Berry M.F., Worni M., Pietrobon R., D'Amico T.A., Akushevich I. (2013). Variability in the treatment of elderly patients with stage IIIA (N2) non-small-cell lung cancer. J Thorac Oncol.

[21] Darling G., Malthaner R., Dickie J. (2014). Quality indicators for non-small cell lung cancer operations with use of a modified Delphi consensus process. Ann Thorac Surg.

[22] Driessen E.J.M., Schulkes K.J.G., Dingemans A.C. (2018). Patterns of treatment and survival among older patients with stage III non-small cell lung cancer. Lung Cancer.

[23] Enewold L., Thomas A. (2016). Real-world patterns of EGFR testing and treatment with erlotinib for non-small cell lung cancer in the United States. PLoS One.

[24] Furqan M., Tien Y.Y., Schroeder M.C. (2018). Lobar versus sub-lobar surgery for pulmonary typical carcinoid, a population-based analysis. J Thorac Dis.

[25] Jakobsen E., Green A., Oesterlind K., Rasmussen T.R., Iachina M., Palshof T. (2013). Nationwide quality improvement in lung cancer care: the role of the Danish Lung Cancer Group and Registry. J Thorac Oncol.

[26] Kapadia N.S., Valle L.F., George J.A. (2017). Patterns of treatment and outcomes for definitive therapy of early stage non-small cell lung cancer. Ann Thorac Surg.

[27] Khare S.R., Batist G., Bartlett G. (2016). Identification of performance indicators across a network of clinical cancer programs. Curr Oncol.

[28] Nilsson J., Berglund A., Bergström S., Bergqvist M., Lambe M. (2017). The role of comorbidity in the management and prognosis in nonsmall cell lung cancer: a population-based study. Acta Oncol.

[29] Sandelin M., Berglund A., Sundström M. (2015). Patients with non-small cell lung cancer analyzed for EGFR: adherence to guidelines, prevalence and outcome. Anticancer Res.

[30] Scottish Cancer Taskforce National Cancer Quality Steering Group (2017).

[31] Shen C., Kehl K.L., Zhao B., Simon G.R., Zhou S., Giordano S.H. (2017). Utilization patterns and trends in epidermal growth factor receptor (EGFR) mutation testing among patients with newly diagnosed metastatic lung cancer. Clin Lung Cancer.

[32] Sher D.J., Liptay M.J., Fidler M.J. (2014). Prevalence and predictors of neoadjuvant therapy for stage IIIA non-small cell lung cancer in the National Cancer Database: importance of socioeconomic status and treating institution. Int J Radiat Oncol Biol Phys.

[33] Skov B.G., Høgdall E., Clementsen P. (2015). The prevalence of EGFR mutations in non-small cell lung cancer in an unselected Caucasian population. APMIS.

[34] Stirling R., Evans S.M., McLaughlin P. (2014). The Victorian Lung Cancer Registry Pilot: improving the quality of lung cancer care through the use of a disease quality registry. Lung.

[35] Tsai H.Y., Chung K.P., Kuo R.N.C. (2018). Impact of targeted therapy on the quality of end-of-life care for patients with non-small-cell lung cancer: a population-based study in Taiwan. J Pain Symptom Manage.

[36] Vrijens F., Verleye L., Gendt C.D. (2016).

[37] Wang X., Su S., Li S. (2017). Development of quality indicators for non-small cell lung cancer care: a first step toward assessing and improving quality of cancer care in China. BMC Cancer.

[38] Wu C.Y., Hu H.Y., Pu C.Y. (2011). Pulmonary tuberculosis increases the risk of lung cancer: a population-based cohort study. Cancer.

[39] Harris P.A., Taylor R., Minor B.L. (2019). The REDCap consortium: building an international community of software platform partners. J Biomed Inform.

[40] Bray F., Znaor A., Cueva P. (2014).

[41] Ferlay J., Burkhard C., Whelan S., Parkin D.M. (2005).

[42] Brierley J., Gospodarowicz M., Wittekind C. (2016). TNM Classification of Malignant Tumours.

[43] Sankaranarayanan R., Ramadas K., Qiao Y.L. (2014). Managing the changing burden of cancer in Asia. BMC Med.

[44] Barber S., Lorenzoni L., Ong P., WHO Centre for Health Development (Kobe, Japan) (2019). Price Setting and Price Regulation in Health Care: Lessons for Advancing Universal Health Coverage.

[45] He J., Pericone C.D., Vanderpoel J. (2022). Real-world patient characteristics, treatment patterns, and mutation testing patterns among US patients with advanced non-small cell lung cancer Harboring EGFR mutations. Adv Ther.

[46] Kerr K.M., Bibeau F., Thunnissen E. (2021). The evolving landscape of biomarker testing for non-small cell lung cancer in Europe. Lung Cancer.

[47] Kimman M., Jan S., ACTION Study Group (2015). Catastrophic health expenditure and 12-month mortality associated with cancer in Southeast Asia: results from a longitudinal study in eight countries. BMC Med.

[48] Reungwetwattana T., Oranratnachai S., Puataweepong P., Tangsujaritvijit V., Cherntanomwong P. (2020). Lung cancer in Thailand. J Thorac Oncol.

[49] Ramadani R.V., Thabrany H., Putri A.E. (2018). Inequities of access, utilization and clinical outcome of lung cancer in Indonesia. J Global Oncol.

[50] Rajadurai P., How S.H., Liam C.K., Sachithanandan A., Soon S.Y., Tho L.M. (2020). Lung cancer in Malaysia. J Thorac Oncol.

[51] Ang Y.L.E., Chia P.L., Chua K.L.M. (2021). Lung cancer in Singapore. J Thorac Oncol.

[52] Parkin D.M. (2008). The role of cancer registries in cancer control. Int J Clin Oncol.

[53] Boyle P., Parkin D.M. (1991).

[54] Soerjomataram I., Bardot A., Aitken J. (2021). Impact of the COVID-19 pandemic on population-based cancer registry. Int J Cancer.

[55] Kruk M.E., Gage A.D., Arsenault C. (2018). High-quality health systems in the Sustainable Development Goals era: time for a revolution. Lancet Global Health.

[56] Cortes J., Perez-García J.M., Llombart-Cussac A. (2020). Enhancing global access to cancer medicines. CA Cancer J Clin.

[57] (2021). WHO model list of essential medicines-22nd list. https://www.who.int/publications-detail-redirect/WHO-MHP-HPS-EML-2021.02.

[58] Cherny N.I., Dafni U., Bogaerts J. (2017). ESMO-Magnitude of Clinical Benefit Scale version 1.1. Ann Oncol.

[59] Eniu A., Cherny N.I., Bertram M. (2019). Cancer medicines in Asia and Asia-Pacific: what is available, and is it effective enough?. ESMO Open.

[60] Gatellier L., Matsuda T., Sabapathy K. (2020). An Asian body to tackle cancers in Asia—The Asian National Cancer Centers Alliance. Asian Pac J Cancer Prev.

[61] Moore M.A. (2014). Cancer control programs in East Asia: evidence from the international literature. J Prev Med Public Health.

[62] Smeltzer M.P., Wynes M.W., Lantuejoul S. (2020). The International Association for the Study of Lung Cancer Global Survey on Molecular Testing in Lung Cancer. J Thorac Oncol.

